# Supplementary material for: COVID-19 symptoms predictive of healthcare workers’ SARS-CoV-2 PCR results
Source: PLoS One. 2020 Jun 26;15(6):e0235460. doi: 10.1371/journal.pone.0235460 (PMC7319316; doi:10.1371/journal.pone.0235460)
Supplement: S1 File — (PDF) [file pone.0235460.s001.pdf]

[redacted] Occupational Health COVID-19/Influenza like Illness Triage Form

EMPLOYEE

Name ____________ DOB ____________ Cell Phone ____________ Date/Time of Employee Contact ____________

Role ____________ Department ____________ Supervisor/Contact info ____________

Have you called the COVID hotline before? Y N Date ____________

Currently: ____ At work ____ At home

Last Shift Worked: ____________

EXPOSURE

___ Close contact (< 6 ft for ≥ 10 min) with PUI or COVID+ w/out adequate PPE:

Patient Employee Household Community

___ Travel within last 2 weeks *(dates/locations)*:

Previous tests for COVID-19?

Result ____________ Date ____________

Result ____________ Date ____________

If patient, employee, or household exposure:

Self:	PPE?	Gown	Face Shield	Gloves	
	Mask usage?	N95	Surgical	Cloth	None
Contact:	Mask usage?	N95	Surgical	Cloth	None

Any aerosolizing testing/procedures? Y N

Household Contacts (include ages/symptoms):

SYMPTOMS

Sx Start Date: ____________

Performing job duties while symptomatic? Y N

____ Fever (subjective OR measured ≥ 99.5 °F)
____ Temp measured ______°F
____ Cough
____ Shortness of breath
____ Myalgia (muscle pains)
____ Malaise (fatigue/feeling tired)
____ Sore throat
____ Runny nose / nasal congestion
____ Nausea | Vomiting | Diarrhea (check & circle)
____ Rash
____ Loss of smell or taste
____ Headache
____ Other:

____________ RN Name ____________ Signature ____________ Date

MANAGEMENT

____ Cleared to RTW ☐ No restrictions ________

☐ With restrictions: ____ Mask ____ Avoid contact with high risk patients

____ Cleared to RTW + COVID-19 testing (asymptomatic employees)

____ OOW + COVID-19 testing

____ OOW: If remains asymptomatic, test 7 days after household contact diagnosed (if can isolate)
If develops symptoms, call back for testing

____ OOW: ____________

Dates:

OCC HEALTH RTW: ________ or TODAY

OCC HEALTH PLACED OOW: TODAY

7D AFTER POS. CONTACT Dx: ________

____________ MD Name ____________ Signature ____________ Date

GUIDANCE

____ Anticipatory guidance on self-monitoring, hand hygiene, droplet precaution, preventing transmission

____ Self-isolation until Occ Health notifies about test result

____ Able to self-quarantine for prescribed period of time from last contact

______ Employee updated on management & guidance
RN initials

ADMINISTRATIVE

____ Manager informed

____ Entered into spreadsheet

______ Initials
