# Supplementary material for: COVID-19 symptoms predictive of healthcare workers’ SARS-CoV-2 PCR results
Source: PLoS One. 2020 Jun 26;15(6):e0235460. doi: 10.1371/journal.pone.0235460 (PMC7319316; doi:10.1371/journal.pone.0235460)
Supplement: S1 Table — (DOCX) [file pone.0235460.s002.docx]

| **HCW Age/ Sex** | **Days Symptomatic before Triage & PCR Test** | **Initial Triage Symptoms** | **Days Elapsed from Onset to Repeat Triage & Test** | **Repeat Triage Symptoms** |
| --- | --- | --- | --- | --- |
| 29/F | 1 | fever (38.6°C)  cough  myalgia  diarrhea | 3 | fever (38°C) with antipyretics  sore throat  cough |
| 34/F | 1 | myalgia  malaise  sore throat  nasal symptoms headache | 15 | diarrhea  headache |
| 23/M | 1 | cough  nasal symptoms  headache | 17 | anosmia/ageusia |
| 23/F | 1 | cough | 19 | cough  myalgia  malaise  sore throat  headache |
| 26/F | 2 | fever (37.7°C)  cough  myalgia  malaise  sore throat  nasal symptoms  headache | 8 | nasal symptoms  anosmia/ageusia |
| 56/F | 5 | myalgia  malaise  nasal symptoms  GI symptoms | 11 | fever (38.7°C) sore throat |
| 47/M | 6 | cough  myalgia  malaise  sore throat  nasal symptoms | 13 | fever (37.7°C)  cough  myalgia  malaise  nasal symptoms  GI symptoms |
| 28/F | 8 | cough  sore throat | 28 | fever (40.0°C)  shortness of breath  myalgia  malaise  GI symptoms  headache |
| 58/F | 14 | fever  cough  malaise  nasal symptoms  GI symptoms | 17 | Cough  GI symptoms anosmia/ageusia headache |

Age in year-old. Sex: F denotes female; M denotes male.
