# Supplementary material for: COVID-19 symptoms predictive of healthcare workers’ SARS-CoV-2 PCR results
Source: PLoS One. 2020 Jun 26;15(6):e0235460. doi: 10.1371/journal.pone.0235460 (PMC7319316; doi:10.1371/journal.pone.0235460)
Supplement: Attachment — Submitted filename: Comments_Kales_etal_Pone_June-2020.doc [file pone.0235460.s003.doc]

June 6, 2020

Manuscript PONE-D-20-13207

Comments on manuscript:

‘COVID-19 Symptoms Predictive of Healthcare Workers’ SARS-CoV-2 PCR Results’

This manuscript concerns a study among health care workers (HCW) and factors that are associated with testing positive for SARS-2 coronavirus. The data are sound and interesting and may be helpful to determine efficient testing strategies.

Some points need to be considered and adjusted before this paper is suitable for publication in PlosOne.

**General comments**:

-The Discussion section is rather long and may be shortened by removing redundant statements. For example page 22, second paragraph: this overlaps with other statements in the Discussion.

-In the conclusions of the abstract and at the end of the manuscript, is stated that anosmia/ageusia and fever were the two strongest predictors of positive assays. In Table 1 and Table 4 however, it is clear that also myalgia is significantly associated with positive SARS-2 test results. This is also mentioned in the discussion. Why is this not mentioned in the conclusions in both abstract and discussion?

-In addition: please mention as early as possible in the manuscript that ‘anosmia/ageusia’ means ‘smell/taste’ since these are medical terms with which not all readers are familiar.

-Could the authors speculate on the usefulness of their data for the general population? So would these indicators for a positive SARS-2 test also be true for non-HCW?

**Specific comments**

Abstract

-Page 3, 5^th^ line: please insert ‘was’ between ’but’ and ‘associated’.

Methods

-Page 7/8: here the RT-PCR assays are mentioned and it is stated that results were either ‘positive’ or ‘negative’. Does this mean that there were no ‘indeterminate’ results? That is a bit hard to believe. If there were indeed ‘indeterminate’ results, how were these dealt with?

-Page 8, Data collection, second paragraph: please replace ‘Absence of a gold standard’ by ‘Because there is no reference diagnostic test’, since the term ‘gold standard’ is not appropriate. The same point for page 24, second paragraph, in the Discussion.

Results

Table 3, legend: here ‘loss of smell/taste’ is mentioned instead of ‘anosmis/ageusia’. Please be consistent (but also adjust according to the general comment above). Same comment for Table 4, legend.

Supplementary Table 1: Two columns ‘Initial test result’ and ‘Repeat test result’ seem superfluous, since for all HCW in this S1 Table the initial test results were negative and the ‘Repeat test results’ were positive, since this was the objective of this Table. I suggest removing these 2 columns and to provide a legend for this Table in which is indicated that for all HCW initial test results were negative and the repeat test results were positive.

-Page 19, last sentence of the Results: please replace ‘requiring’ by ‘required’ (twice), ‘needing’ by ‘needed’ and ‘dying’ by ‘died’.

Discussion

Page 19, last sentence: a total of 16% is mentioned, but in the abstract this is 14%. Probably the ‘false negatives’ are involved here. Please mention this more explicitly.
